# Multi-label classification with XGBoost for metabolic pathway prediction

**DOI:** 10.1186/s12859-024-05666-0

**Published:** 2024-02-01

**Authors:** Hyunwhan Joe, Hong-Gee Kim

**Affiliations:** 1 Biomedical Knowledge Engineering Lab., Seoul National University, Seoul, Republic of Korea; 2 School of Dentistry and Dental Research Institute, Seoul National University, Seoul, Republic of Korea

**Keywords:** Metabolic pathway prediction, BioCyc, XGBoost

## Abstract

****Background**:**

Metabolic pathway prediction is one possible approach to address the problem in system biology of reconstructing an organism’s metabolic network from its genome sequence. Recently there have been developments in machine learning-based pathway prediction methods that conclude that machine learning-based approaches are similar in performance to the most used method, PathoLogic which is a rule-based method. One issue is that previous studies evaluated PathoLogic without taxonomic pruning which decreases its performance.

****Results**:**

In this study, we update the evaluation results from previous studies to demonstrate that PathoLogic with taxonomic pruning outperforms previous machine learning-based approaches and that further improvements in performance need to be made for them to be competitive. Furthermore, we introduce mlXGPR, a XGBoost-based metabolic pathway prediction method based on the multi-label classification pathway prediction framework introduced from mlLGPR. We also improve on this multi-label framework by utilizing correlations between labels using classifier chains. We propose a ranking method that determines the order of the chain so that lower performing classifiers are placed later in the chain to utilize the correlations between labels more. We evaluate mlXGPR with and without classifier chains on single-organism and multi-organism benchmarks. Our results indicate that mlXGPR outperform other previous pathway prediction methods including PathoLogic with taxonomic pruning in terms of hamming loss, precision and F1 score on single organism benchmarks.

****Conclusions**:**

The results from our study indicate that the performance of machine learning-based pathway prediction methods can be substantially improved and can even outperform PathoLogic with taxonomic pruning.

## Introduction

A fundamental prerequisite in comprehending an organism’s metabolism is the realization of an encompassing model of the metabolic interactions that occur in the organism [1]. An example of such a model is a Pathway/Genome Database (PGDB) that describes an organism’s genes, proteins and metabolic and regulatory networks [2]. Initially, PGDBs were constructed through literature-based manual curation but this approach was not scalable [3]. This led to hybrid approaches where PGDBs are initially generated then refined through manual curation afterwards [4].

**Fig. 1:**
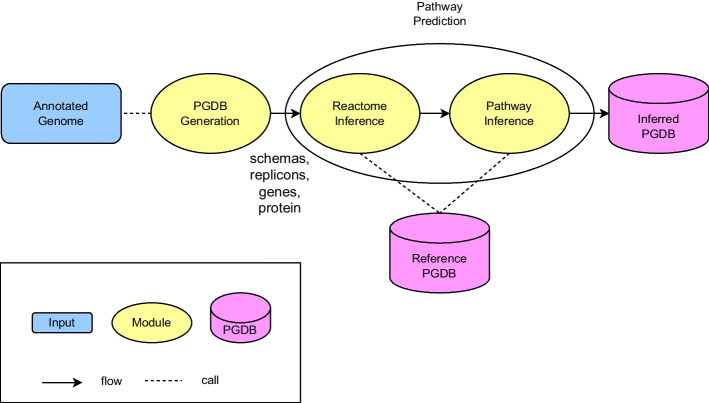
Workflow of PGDB creation

The PGDB creation workflow used by Pathway Tools [2], a software environment that is used to create and manage PGDBs, consists of two main steps with additional post-processing steps afterwards which can be seen in Fig. 1. The first step is the PGDB generation step where the schema, replicons, genes and proteins of a PGDB are generated from an organism’s annotated genome. The next step is the pathway prediction step which is divided into two sub-steps. The first sub-step performs reactome inference where the set of enzyme-catalyzed metabolic reactions occurring in an organism are predicted. The second sub-step is pathway inference where, based on the predicted reactome, the pathways occurring in the organism are predicted. Only metabolic pathways are predicted instead of other types of biochemical pathways such as signaling pathways. Metabolic pathway prediction in the literature commonly refers to predicting either the metabolic pathways that a molecule is associated with [5–7] or the metabolic pathways occurring in an organism based on its annotated genome [1, 8, 9]. This work will focus on the latter and assumes that the reactome is already inferred and provided. Lastly, pathway prediction can also be differentiated into predicting pathways from a reference database and predicting unobserved novel pathways (pathway discovery) [1] and this work focuses on the former.

PathoLogic is a pathway prediction algorithm developed by SRI International that is used by Pathway Tools. PathoLogic predicts metabolic pathways in MetaCyc [10], a curated reference metabolic pathway database, from an organism’s annotated genome. It assigns scores to each metabolic pathway in MetaCyc, where a higher score reflects a higher likelihood that the pathway is present in the target organism. Afterwards, the decision to include or reject the pathway is completed through a sequence of defined rules [8]. While PathoLogic has gone through several iterations and updates to improve its accuracy, it has several limitations. One limitation is that since the rules defined are hard-coded, it makes the algorithm relatively inflexible to maintain and extend. Another limitation is that the pathway scoring system is ad-hoc and does not reflect actual mathematical probabilities.

As a response to these limitations, Dale et al. [1] introduced the first study that evaluated multiple machine learning-based metabolic pathway prediction methods. Their results demonstrated that machine learning methods were able to perform as well as PathoLogic with the best performing ML-based approach achieving a small improvement over PathoLogic. Despite the promising results from the study, PathoLogic is still used as the main engine for Pathway Tool’s prediction algorithm. Recently, there has been several studies which updated the pioneer study with new datasets, features and methodologies [11–13].

One of the studies mlLGPR [11], made a novel contribution of modeling the prediction task as multi-label classification compared to other studies which modeled it as binary classification. Multi-label classification is where more than one class label can be predicted which differs from traditional classification where only one label is predicted [14]. Modeling the prediction task as multi-label classification allowed the training dataset used in mlLGPR to be more compact allowing for more organisms to be used for training. For example, Aljarbou et al. [12] has 4979 instances covering 20 organisms and DeepRF [13] had 172,380 instances covering 60 organisms. mlLGPR’s multi-label modeling allows for its dataset to be smaller with 15,000 instances but is able to cover 15,000 organisms. mlLGPR uses a binary relevance approach [15] where the multi-label learning process is divided into independent binary classifiers for each pathway label allowing for the possibility of parallel training. What also differentiated mlLGPR with other pathway prediction studies such as [1, 12, 13] is that for their evaluation methodology they used a completely separate evaluation dataset which they did not use for training and hyperparameter tuning. Another novel contribution from the mlLGPR study was that it was the first machine learning-based pathway prediction method to be evaluated also on multi-organism genomes such as symbionts and microbiomes. The evaluation results from mlLGPR were also similar to other studies on single-organism genomes showing similar performance to PathoLogic.

A limitation of previous machine learning-based metabolic pathway prediction methods was that the feature engineering task involving designing and testing features was a time consuming task. As a response to this limitation, representational learning approaches [16] such as pathway2vec [9] and triUMPF [17] were introduced to generate features to be used for prediction. While the research direction and results from the two studies are promising, they shared similar problems with mlLGPR in their evaluation methodology for single organism genomes. The common issue is that PathoLogic is evaluated without using taxonomic pruning. MetaCyc pathways can be assigned a taxonomic range for which they can occur and PathoLogic utilizes these ranges when deciding on whether to include or reject a pathway. Taxonomic pruning was introduced to improve the performance of PathoLogic by removing false positives [8]. While the mlLGPR study acknowledges that PathoLogic was evaluated without using taxonomic pruning for the single-organism benchmark it does not give the reason for not applying it when it improves performance. This is an issue because evaluating PathoLogic without using taxonomic pruning for single organism genomes can lead to potentially lower results which can be misleading as a benchmark.

In this study, we provide three contributions to the problem of metabolic pathway prediction from annotated genomes. The first contribution is that we evaluate PathoLogic with taxonomic pruning on the single organism prediction benchmark to provide a more accurate pathway prediction benchmark. Our results show that PathoLogic with taxonomic pruning showed a significant increase on the four evaluation metrics for the majority of the single organism datasets. In addition, we observed that the evaluation datasets introduced in the mlLGPR study shares characteristics of tabular datasets with its mixed feature data types. Recent studies have shown that tree ensemble models such as XGBoost tend to outperform deep learning prediction models when applied to tabular datasets [18, 19]. With these observations, for our second contribution we introduce a XGBoost-based pathway prediction method termed mlXGPR based on the multi-label classification prediction framework introduced by mlLGPR and evaluate it on single organism and multi-organism benchmark datasets. For our third contribution, we further improve on mlXGPR by using classifier chains [15] which uses predictions from previous classifiers as features for future classifiers to take advantage of correlations between labels. We introduce a ranking mechanism that allows for higher performing classifiers to be earlier in the chain while lower performing classifiers are put later in the chain so they can utilize previous predictions. With these improvements, mlXGPR outperformed the other prediction methods including PathoLogic with taxonomic pruning for three of the evaluation metrics hamming loss, precision and F1 score on the single organism benchmarks.

## Methods

**Fig. 2:**
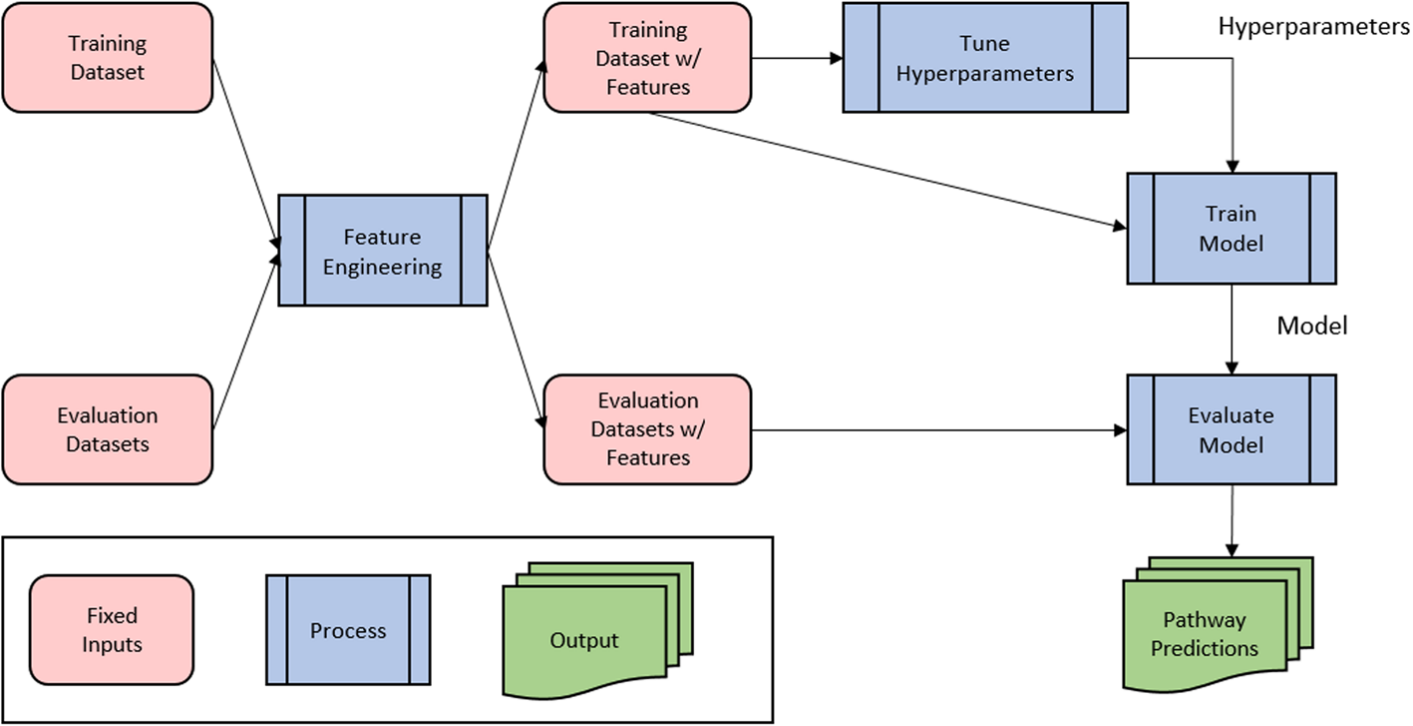
mlXGPR workflow

The workflow for mlXGPR is similar to the multi-label classification for metabolic pathway prediction workflow introduced in the mlLGPR study. The first step is the feature engineering step which takes the training and evaluation datasets and transforms them into feature vectors. The mlLGPR study introduced five different feature groups which are enzymatic reaction abundance (AB), reaction evidence (RE), pathway evidence (PE), pathway commons (PC) and possible pathways (PP) where AB is the main feature group that can be combined with other feature groups. After the training dataset is transformed into feature vectors, we use k-fold cross validation and grid search to tune the hyperparameters of our prediction model. Once the hypermeters are chosen for the final prediction model, the whole training dataset is then used for training the model. The trained model is then evaluated on the benchmark datasets and then can be deployed to predict new datasets. One difference between mlLGPR and mlXGPR is that mlXGPR uses XGBoost as the prediction model instead of logistic regression as used in mlLGPR. Another difference is that mlLGPR does not use cross-validation for hyperparameter tuning but used one split to tune its hyperparameters. The workflow for mlXGPR can be seen in Fig. 2.

### Definitions and problem formulation

In this study, we will use the conventions introduced in the mlLGPR study [11]. All vectors are column vectors which are denoted by boldface lowercase letters (e.g. ${{\textbf {x}}}$) while matrices are denoted by boldface uppercase letters (e.g. ${{\textbf {X}}}$). A subscript character to a vector, ${{\textbf {x}}}_i$, denotes the *i*-th element of ${{\textbf {x}}}$ while a superscript, ${{\textbf {x}}}^{i}$, denotes an index to a sample. In addition, calligraphic letters (e.g. ${\mathcal {S}}$) are used to represent sets and |.| will be used to denote set cardinality. A multi-label pathway dataset consisting of *n* samples can be defined as ${\mathcal {S}} = \{ ({{\textbf {x}}}^{i},{{\textbf {y}}}^{i}) : 1 \leqslant i \leqslant n \}$. ${{\textbf {x}}}^i$ is a vector that corresponds to the abundance of each enzymatic reaction *e*, which is an element of the set ${\mathcal {E}}=\{e_1,e_2,...,e_r\}$, having *r* possible reactions. The abundance of an enzymatic reaction $e_l^{i}$, for a sample *i* can be defined as $a_l^{i}\in {\mathbb {R}}_{\ge 0}$. The class labels ${{\textbf {y}}}^{i}=[y_1^{i},...,y_t^{i}]\in \{-1,1\}^t$ is a vector of size *t*. Its elements correspond to pathway labels derived from a reference pathway database ${\mathcal {Y}}$. A sample for the multi-label pathway dataset used can be seen in Table 1

${\mathcal {X}}={\mathbb {R}}^r$ is defined as the *r*-dimensional input space. Each sample ${{\textbf {x}}}^{i} \in {\mathcal {X}}$ is transformed into an *m*-dimensional vector by a transformation function $\Phi :{\mathcal {X}} \rightarrow {\mathbb {R}}^m$. The transformation function is obtained from the Feature engineering process (see Section Features engineering). In summary, the metabolic pathway prediction task can be defined as given a multi-label dataset ${\mathcal {S}}$, learn a hypothesis function $f : \Phi ({{\textbf {x}}}^{i})\mapsto 2^{|{\mathcal {Y}}|}$, such that it can classify metabolic pathway labels accurately for an unseen sample ${{\textbf {x}}}^*$.

**Table 1:** Sample of multi-label pathway dataset

*Input enzymatic reaction abundances*				
EC-1	EC-1.1	...	EC-6.6.1.1	EC-6.6.1.2
2	9	...	1	0
*Output presence of pathways*				
VALSYN	ARG-PRO	...	PWY-7081	PW-721
1	0	...	1	0

The number of pathways is independent from the number of enzymatic reactions

### Feature engineering

Five types of feature vectors were designed and introduced in the mlLGPR study [11]. Each feature vector is created through 5 transformation sub-processes (1) enzymatic reactions abundance ($\phi ^{ab}$), (2)- reactions evidence ($\phi ^{re}$), iii)- pathways evidence ($\phi ^{pe}$), iv)- pathway common ($\phi ^{pc}$) and v)- possible pathways ($\phi ^{pp}$). The enzymatic reaction abundance transformation maps to a *r*-dimensional vector that denotes the total occurrence of each enzymatic reaction in an organism. Each enzymatic reaction is identified by its Enzyme Commission (EC) number [20]. The reaction evidence transformation maps to a vector that represents the properties of the enzymatic reactions for each sample. The pathway evidence transformation maps to a vector whose features expands on core PathoLogic rules to also include enzyme presence, pathway gaps, network connectivity and etc. The possible pathway transformation maps to a vector which holds for each pathway two representations. The first is a boolean representation, whether each pathway is present or not, from enzymatic reaction information, and is decided by a user-defined threshold. The second is a numeric representation which represents the probabilities for each pathway whether they are present or not based off enzymatic reaction information. Each transformation maps a sample to a different vector which are concatenated into a *m*-dimensional feature vector $\Phi ({{\textbf {x}}}^{(i)})=[\phi ^{ab}({{\textbf {x}}}^{(i)}),\phi ^{re}({{\textbf {x}}}^{(i)}),\phi ^{pe}({{\textbf {x}}}^{(i)}), \phi ^{pc}({{\textbf {x}}}^{(i)}), \phi ^{pp}({{\textbf {x}}}^{(i)})]$. The number of features for each feature group can be seen in Table 2.

**Table 2:** Number of features for each feature group

Feature group	Number of features
Enzymatic Reaction Abundance	3650
Reaction Evidence	68
Pathway Evidence	32
Pathway Commons	3650
Possible Pathways	5052

### Prediction model and multi-label learning process

XGBoost is a machine learning algorithm that utilizes gradient boosted decision trees [21] where each tree is trained to predict the pseudo-residuals of the previous tree based on a pre-defined objective function [22]. One of the key factors in XGBoost’s success and popularity is innovations in scalability such as optimizations in handling sparse data, weighted quantile sketch calculations and parallel/distributed computing [23]. Recently, XGBoost version 1.6 started to provide native support for multi-label classification which allows for the efficient training of classifiers on many class labels. Before this addition, studies used outside libraries such as scikit-multilearn [24–26] or sklearn.MultiOutputClassifier for multi-label classification [27].

To define the binary relevance approach we will introduce conventions used here [15]. ${\mathcal {Y}}=\{\lambda _1,\lambda _2,...,\lambda _t\}$ is the label space which consist of *t* class labels. The set of relevant labels $Y^{i} \subseteq {\mathcal {Y}}$ for a given ${{\textbf {x}}}^{i}$ can be defined as $Y^{i} =\{\lambda _j | y^{i}_j = +1, 1 \leqslant j \leqslant t\}$. Binary relevance breaks down the multi-label learning problem into *t* independent binary classification problems where each problem corresponds to a class label $\lambda _j$. First, a binary training set ${\mathcal {S}}_j$ is derived from the multi-label pathway dataset ${\mathcal {S}}$ according to Eq. (1):

1 $$\begin{aligned} {\mathcal {S}}_j=\{({{\textbf {x}}}^{i}, y^{i}_j) : 1 \leqslant i \leqslant n \} \end{aligned}$$

Afterwards, a binary classifier $g_j : {\mathcal {X}} \mapsto {\mathbb {R}}$ is induced from ${\mathcal {S}}_j$ through the application of a binary learning algorithm ${\mathcal {B}}$. When an unseen instance ${{\textbf {x}}}^*$ is given as input, the binary relevance procedure outputs its relevant label set $Y^*$ which is determined by the output of each binary classifier as in Eq. (2):

2 $$\begin{aligned} Y^*=\{ \lambda _j | g_j({{\textbf {x}}}^*) > 0, 1 \leqslant j \leqslant t \} \end{aligned}$$

Algorithm 1 summarizes the process for binary relevance.

To define a classifier chain we will use the same conventions introduced earlier defining binary relevance, in addition to conventions in [15]. $\pi$ is the permutation that specifies a chaining order over the class labels. The binary training set ${\mathcal {S}}_{\pi (j)}$ for the *j*th class label $\lambda _{\pi (j)}$ is derived according to Eq. (3):

3 $$\begin{aligned} {\mathcal {S}}_{\pi (j)}=\{([{{\textbf {x}}}^*,y^i_{\pi (1)},...,y^i_{\pi (j-1)}],y^i_{\pi (j)}|1 \leqslant i \leqslant n)\} \end{aligned}$$

Afterwards, a binary classifier $g_{\pi (j)}: {\mathcal {X}} \times \{-1,+1\}^{j-1} \mapsto {\mathbb {R}}$ can be induced by applying a binary algorithm ${\mathcal {B}}$ to the binary training set ${\mathcal {S}}_{\pi (j)}$. For a given unseen instance ${{\textbf {x}}}^*$, the predicted binary assignment $\eta ^{{{\textbf {x}}}^*}_{\pi (j)}\in \{-1,+1\}$ for label $\lambda _{\pi (j)}$ is determined as in Eq. 4:

4 $$\begin{aligned} \eta ^{{{\textbf {x}}}^*}_{\pi (1)}&= \text {sign}[g_{\pi (1)}({{\textbf {x}}}^*)], \nonumber \\ \eta ^{{{\textbf {x}}}^*}_{\pi (j)}&= \text {sign}[g_{\pi (j)}([{{\textbf {x}}}^*,\eta ^{{{\textbf {x}}}^*}_{\pi (1)},...,\eta ^{{{\textbf {x}}}^*}_{\pi (j-1)}])] \end{aligned}$$

where sign$[\cdot ]$ represents the sign function. The relevant label set $Y^*$ is derived according to Eq. (5):

5 $$\begin{aligned} Y^*=\{ \lambda _{\pi (j)} | \eta ^{{{\textbf {x}}}^*}_{\pi (j)}=+1, 1 \leqslant j \leqslant t \} \end{aligned}$$

Algorithm 2 summarizes the process for a classifier chain.

One strategy to combat the randomness by the permutation ordering $\pi$ is to use an ensemble of classifier chains with random permutations [15]. The outputs from all classifier chains in the ensemble can then be aggregated to determine the final prediction. We decided against an ensemble approach because of the large amount of labels and the slower training speed of classifier chains. Instead, we determined the chain order by ranking in descending order the performance of each classifier in a multi-label classifier that uses binary relevance. The reason we determined the order based on a multi-label classifier using binary relevance is because each classifier is independent of the other classifiers. The multi-label training ${\mathcal {S}}$ is split into a training set ${\mathcal {S}}^{'} \subset {\mathcal {S}}$ and valid set ${\mathcal {V}} \subset {\mathcal {S}}$. A multi-label classifier ${\mathcal {G}}$ using binary relevance is trained on ${\mathcal {S}}^{'}$. ${{\textbf {Y}}}^{{\mathcal {V}}}$ is the relevant labels obtained from ${\mathcal {V}}$ while ${\hat{\mathbf{{{Y}}}}}^{{\mathcal {V}}}$ is the predicted labels from ${\mathcal {G}}$ for ${\mathcal {V}}$. Let *scores* be denoted as an empty list. The performance for each classifier in the multi-label classier ${\mathcal {G}}$ is obtained through a metric from the *j*-index of the transpose of ${{\textbf {Y}}}^{{\mathcal {V}}}$ and ${\hat{\mathbf{{{Y}}}}}^{{\mathcal {V}}}$. The metric that was used in the study is the F1-score. The metric score is then appended to *scores* and this process is iterated until the metric score for each label is obtained. Finally, *scores* is sorted in descending order to determine the permutation order $\pi$. Lower performing labels are put later in the chain so they can utilize potential correlations between earlier labels to provide higher quality predictions. Algorithm 1 summarizes the ranking process to determine the chain order.

**Algorithm 1:**
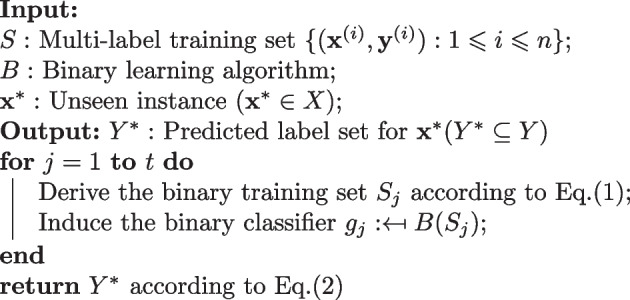
Binary relevance [15]

**Algorithm 2:**
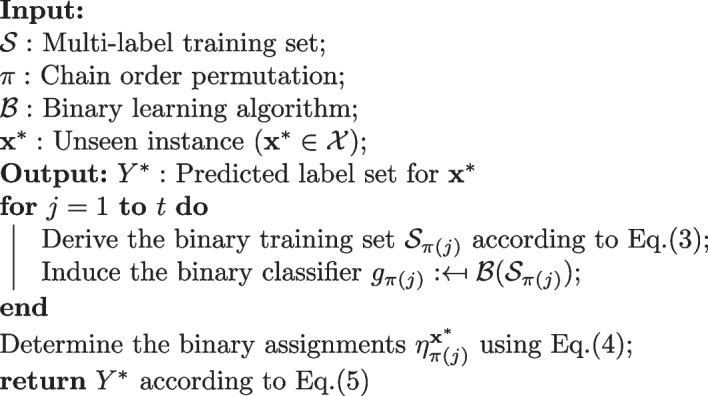
Classifier chain [15]

**Algorithm 3:**
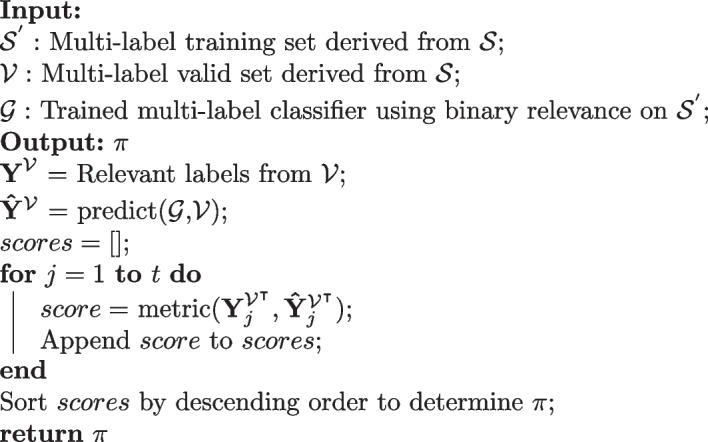
Determine chain order by ranking

### Experimental setup

In this section, we describe the experimental setup to evaluate mlXGPR’s pathway prediction performance across multiple datasets including single and multi-organisms. For training, we used the corrupted synthetic dataset Synset-2 that was constructed and used for training in the mlLGPR study. Synset-2 was constructed from MetaCyc version 21 and contains 2526 metabolic pathways and 3650 enzymatic reactions including incomplete ones such as EC 1.2.3-. The dataset was generated by randomly selecting pathways for each synthetic sample based on the Poisson distribution with mean value equal to 500. The corruption process is done by randomly retaining/inserting/removing enzymatic reactions from each selected pathway based on earlier defined constraints. The dataset was corrupted to reflect errors that could occur from upstream data analysis on experimental data. Synset-2 consists of 15,000 synthetic samples as can be seen in Table 3.

The single organism golden dataset consists of six Tier 1 PGDBs from BioCyc which are EcoCyc(v21) [28], HumanCyc(v19.5) [4], AraCyc(v18.5) [29], YeastCyc(v19.5), LeishCyc(v19.5) [30] and TrypanoCyc(v18.5) [31] which were used in previous benchmarks [9, 11, 17]. BioCyc is a PGDB Web portal that contains thousands of PGDBs and divides PGDBs into tiers based on the manual curation involved [32]. Tier 1 is the highest quality PGDB in BioCyc and the requirement is at least one person year worth of literture-based curation. LeishCyc and TrypanoCyc are currently Tier 2 but the versions used during the mlLGPR study were Tier 1 at the time when the benchmark dataset was created. Basic statistical information for each PGDB can be seen in Table 3. For the multi-organism benchmark dataset we used the Critical Assessment of Metagenome Interpretation (CAMI) initiative low complexity dataset [33] used in the triUMPF study [17].

**Table 3:** Dataset Statistics

Dataset	Instances	Enzymatic reactions	Pathways
Synset-2	15000	3650	2526
EcoCyc	1	719	307
HumanCyc	1	693	279
AraCyc	1	1034	510
YeastCyc	1	544	229
LeishCyc	1	292	87
TrypanoCyc	1	512	175
CAMI	40	1083	674

mlXGPR’s performance was compared to three representative pathway prediction methods. We evaluated PathoLogic version 22 with and without taxonomic pruning, with the default pathway prediction score cutoff value to showcase the improvements in performance with taxonomic pruning. The default value was used because the User’s Guide for Pathway Tools version 22 mentions that the default value was selected to provide the best trade-off between sensitivity and specificity based on extensive experimentation. While the mlLGPR study used PathoLogic v21 without taxonomic pruning, since version 21 is not available to be downloaded anymore, version 22 was used instead. One difference between the two versions is that PathoLogic v22 predicts pathways from MetaCyc v22 which removed 7 pathways from MetaCyc v21 but we were able to get similar results from version 22 without taxonomic pruning with the results from version 21.

MinPath is another well known pathway prediction method that uses integer programming to predict the minimum set of pathways [34]. We did not include MinPath in our evaluation because it had too many false positives leading to low precision as can be seen in the mlLGPR study. For the representative machine learning-based pathway prediction methods we included both results from the mlLGPR and triUMPF [17] studies. The models from Aljarbou et al. [12] and DeepRF [13] were not used in the evaluation because both models are binary classifiers instead of multi-label and are trained using different datasets making it difficult to accurately compare. In addition, from the best of our knowledge the datasets and source code used in both studies are not open source which make comparing their performances even more difficult.

An ablation test on the five feature groups (AB, RE, PE, PP and PC) was done in the mlLGPR study and a combination of +AB+RE+PE feature groups yielded the highest prediction performance with +AB+RE performing the second highest. mlXGPR does not use the Pathway Evidence (PE) feature group because different PE features are used for each pathway label and XGBoost does not natively support this type of multi-label classification. XGBoost only supports multi-label classification where the features are the same throughout each label. mlXGPR also does not use the Pathway Commons and Possible Pathways feature group because the ablation study in the mlLGPR study suggests that these feature groups seem to decrease performance.

For the performance metrics, we used the Hamming loss [35], precision, recall and F1 score to match the metrics used in the previous studies. mlXGPR uses 6-fold cross validation grid search on the training dataset Synset-2 to determine the optimal hyperparameters for the max depth and number of estimators. We used the Scikit-Learn API for XGBoost and the options for the max depth was {2,4,6,8} and {22,23,24} for the number of estimators. The options for the number of estimators was chosen by pre-testing with early stopping. The final model was trained using all of Synset-2 with max depth set to 4 and the number of estimators set to 22 based on the highest average F1 score from grid search. In addition we also used ’hist’ for the tree method because it was fastest among the other options and all the options had similar results. The ’hist’ option is an approximate tree method similar to the method used in LightGBM [36] which is another well known gradient boosting decision tree method. In addition, classifier chains were implemented using scikit-learn. All tests were conducted on an Ubuntu 20.04 server with dual Intel Xeon CPU E5-2640 v4. Python 3.9, XGBoost 1.7 and scikit-learn 1.2 were used to obtain the experimental results.

## Results

Table 4 shows the pathway prediction performance results for mlXGPR and the four other methods. In terms of the Hamming loss, precision and F1 score, mlXGPR using only abundance features (+AB) and mlXGPR with a ranked classifier chain (+RankChain) outperformed the other methods on all the datasets. mlXGPR+AB outranked mlXGPR+AB+RE on most of the metrics on all the datasets except on TrypanoCyc for recall. Both mlXGPR+Chain and mlXGPR+RankChain uses only abundance features (+AB) since the feature group seems to outperform using both abundance and reaction evidence features (+AB+RE). The difference between the two is the mlXGPR+Chain uses a random order while mlXGPR+RankChain uses a chain whose order was determined by ranking each pathway label by their valid set prediction performance. mlXGPR+RankChain outperformed mlXGPR+Chain on most of the metrics and datasets which suggests that the proposed ranking method helped improve performance. PathoLogic without taxonomic pruning had the highest recall on most of the datasets except on EcoCyc where PathoLogic+Pruning had the highest recall and mlXGPR+RankChain had the highest recall on TrypanoCyc. This difference makes sense because taxonomic pruning is designed to prune pathways whose taxonomic range does not match the target organism’s taxonomic group which improves precision at the cost of recall [8]. PathoLogic without pruning and mlLGPR have similar performance in terms of F1 score but earlier benchmarks from the mlLGPR and triUMPF study failed to include PathoLogic with pruning which can be misleading since pruning improves performance. This can be seen from the results that PathoLogic with pruning outperforms both PathoLogic without pruning and mlLGPR on the majority of metrics and datasets. In summary, all future pathway prediction benchmarks on BioCyc PGDBs should include PathoLogic with taxonomic pruning to provide a more accurate evaluation.

**Table 4:** Performance of each prediction algorithm on six single organism T1 PGDBs. $\downarrow$ indicates that a lower score is better while for $\uparrow$ a higher score is better. The best performing method is bold for each metric. PGDB names have been shortened for readability

Metrics and methods	Eco	Human	Ara	Yeast	Leish	Trypano
*Hamming loss* ($\downarrow$)						
PathoLogic	0.0685	0.0744	0.1124	0.0507	0.0416	0.0669
PathoLogic+Pruning	0.0372	0.0424	0.0649	0.0257	0.0234	0.0530
mlLGPR	0.0804	0.0633	0.1069	0.0550	0.0380	0.0590
triUMPF	0.0435	0.0954	0.1560	0.0649	0.0443	0.0776
mlXGPR+AB	**0.0146**	0.0190	0.0412	**0.0146**	**0.0063**	0.0119
mlXGPR+AB+RE	0.0162	0.0226	0.0447	0.0178	0.0075	0.0131
mlXGPR+Chain	0.0190	0.0190	0.0483	0.0174	0.0075	0.0127
mlXGPR+RankChain	0.0158	**0.0170**	**0.0360**	0.0154	0.0079	**0.0099**
*Precision* ($\uparrow$)						
PathoLogic	0.6626	0.6091	0.6799	0.6517	0.4511	0.5099
PathoLogic+Pruning	0.8105	0.7688	0.8502	0.8106	0.6667	0.6589
mlLGPR	0.6187	0.6686	0.7372	0.6480	0.4731	0.5455
triUMPF	0.8662	0.6080	0.7377	0.7273	0.4161	0.4561
mlXGPR+AB	**0.9963**	**0.9873**	0.9833	**1.0000**	**0.9863**	**0.9739**
mlXGPR+AB+RE	0.9890	0.9744	0.9649	0.9742	0.9474	0.9437
mlXGPR+Chain	0.9675	0.9793	0.9641	0.9793	0.9359	0.9441
mlXGPR+RankChain	0.9819	0.9797	**0.9861**	0.9847	0.9241	0.9573
*Recall* ($\uparrow$)						
PathoLogic	0.8893	**0.9104**	**0.8373**	0.**9476**	**0.9540**	0.8857
PathoLogic+Pruning	**0.9055**	0.8817	0.8235	0.9345	0.6437	0.4857
mlLGPR	0.8827	0.8459	0.7314	0.8603	0.9080	0.8914
triUMPF	0.7590	0.3835	0.3529	0.3319	0.7126	0.6229
mlXGPR+AB	0.8827	0.8387	0.8098	0.8384	0.8276	0.8514
mlXGPR+AB+RE	0.8762	0.8172	0.8078	0.8253	0.8276	0.8629
mlXGPR+Chain	0.8730	0.8459	0.7902	0.8253	0.8391	0.8686
mlXGPR+RankChain	0.8860	0.8638	0.8333	0.8428	0.8391	**0.8971**
*F1 Score* ($\uparrow$)						
PathoLogic	0.7594	0.7299	0.7504	0.7722	0.6125	0.6472
PathoLogic+Pruning	0.8554	0.8214	0.8367	0.8682	0.6550	0.5592
mlLGPR	0.7275	0.7468	0.7343	0.7392	0.6220	0.6768
triUMPF	0.8090	0.4703	0.4775	0.4735	0.5254	0.5266
mlXGPR+AB	**0.9361**	0.9070	0.8882	**0.9121**	**0.9000**	0.9085
mlXGPR+AB+RE	0.9292	0.8889	0.8794	0.8936	0.8834	0.9015
mlXGPR+Chain	0.9178	0.9077	0.8685	0.8957	0.8848	0.9048
mlXGPR+RankChain	0.9315	**0.9181**	**0.9033**	0.9082	0.8795	**0.9263**

We also evaluated mlXGPR’s performance on complex multi-organism genomes such as the CAMI low complexity dataset. MetaPathways v2.5 [37] was used to create the benchmark CAMI environment PGDB (ePGDB) which are PGDBs for microbial communities [38]. MetaPathways utilizes a modified version of PathoLogic for pathway prediction. mlXGPR+Chain was compared with two other pathway prediction methods mlLGPR and triUMPF and the results can be seen in Table 5. PathoLogic was not included in the comparison since MetaPathways uses it to create the ePGDB. The results for mlLGPR and triUMPF were taken from the triUMPF study. triUMPF achieved the lowest Hamming loss 0.0436 and the highest sample average F1 score 0.5864. mlLGPR had the highest sample average recall 0.7827 but lowest sample average precision 0.357 in comparison. mlXGPR+Chain was the opposite with the highest sample average precision 0.8366 but the lowest sample average recall 0.2657 which also contributed to it having the lowest sample average F1 score 0.4019 among the three methods. It is difficult to explain the reason for the opposite behavior between mlXGPR and mlLGPR which is consistent in both single-organism and multi-organism benchmark. One observation we make is that all the datasets including both the training and evaluation datasets are imbalanced where the present pathways labels are only about 1/5 of the total pathway labels as can be seen in Table 3. While this imbalance can’t explain why mlXGPR/mlLGPR is biased towards precision/recall it something that needs to be analyzed further.

One limitation of the CAMI ePGDB as a benchmark is that it is automatically generated using MetaPathways but the predictions have not been curated so it can be said that the results demonstrate more how similar the other prediction methods are with MetaPathways and PathoLogic than their actual prediction performance. One explanation for triUMPF’s higher performance is that it was trained on mostly Tier 3 BioCyc PGDBs instead of Synset-2 like mlLGPR and mlXGPR. Tier 3 PGDBs are generated from PathoLogic without any curation [32], so training a model on Tier 3 PGDBs can be seen as training a model on PathoLogic outputs leading to more similar results with PathoLogic. We tested this by training mlXGPR+Chain on the Tier 3 PGDB training data that triUMPF uses and found a 10% increase in F1 score which can be seen in Table 5. In summary, there is still a lack of highly curated ePGDBs that can be used for multi-organism pathway prediction benchmarks.

**Table 5:** Performance of mlLGPR, triUMPF and mlXGPR on the multi-organism community dataset CAMI

Metrics and methods	mlLGPR	triUMPF	mlXGPR + RankChain	mlXGPR + RankChain (BioCyc)
Hamming loss ($\downarrow$)	0.0975	0.0436	0.0482	**0.0415**
Average Precision ($\uparrow$)	0.3570	0.7027	0.8366	**0.9145**
Average Recall ($\uparrow$)	**0.7827**	0.5101	0.2657	0.3629
Average F1 score ($\uparrow$)	0.4866	**0.5864**	0.4019	0.5185

$\downarrow$ indicates that a lower score is better while for $\uparrow$ a higher score is better. The best performing method is bold for each metric. The sample average is calculated for the average precision, recall and F1 score

## Conclusions

In this study, we introduce a XGBoost-based metabolic pathway prediction method called mlXGPR based on mlLGPR, which introduced an approach that modeled the metabolic pathway inference problem as a multi-label classification problem. mlXGPR was motivated by previous pathway prediction studies in that they were not compared properly with PathoLogic using taxonomic pruning and needed further improvement in performance. In response to this, we attempted to apply XGBoost, a SOTA supervised learning method for tabular data to the problem of multi-label pathway prediction. One potential limitation that mlXGPR has is that it is unable to capture correlations between labels so we also applied classifier chains to mlXGPR so that predictions from earlier classifiers can be used as features by later classifers. We proposed a ranking method to decide the order of the chain by putting lower performing classifiers based on a valid set later in the chain to better utilize previous predictions. We trained mlXGPR and its chained counterpart with tuned hyper-parameters, and compared its performance with three representative metabolic pathway prediction methods on single organism and multi-organism genome benchmark datasets. The results was that mlXGPR with classifier chains outperformed the other methods on three of the four evaluation metrics which are Hamming loss, precision and F1 score for single-organism datasets.

While we were able to improve the performance of machine learning-based pathway prediction methods to outperform PathoLogic using taxonomic pruning, mlXGPR still shares the common issue with mlLGPR in that its performance is reliant on feature information that is manually curated. This is why representational learning-based pathway prediction approaches are promising but currently their performance still need improvement. Another potential direction for future studies in machine learning-based pathway prediction is if the datasets and source code from other studies such as [12] and DeepRF [13] become open, their methodologies can be evaluated on the mlLGPR benchmark datasets. The reverse can also be done with evaluating the methodologies used in mlLGPR, triUMPF and mlXGPR on the different datasets used in these studies. This would allow for a more comprehensive evaluation of the performance of machine learning-based pathway prediction models.

## Data Availability

The data used in the study is available at https://github.com/hyunwhanjoe/mlXGPR/.

## Code availability

he source code used in the study is available at https://github.com/hyunwhanjoe/mlXGPR/.
